# Management information systems for community based interventions to improve health: qualitative study of stakeholder perspectives

**DOI:** 10.1186/s12889-018-6363-z

**Published:** 2019-01-23

**Authors:** Linda Penn, Louis Goffe, Anna Haste, Suzanne Moffatt

**Affiliations:** 1 Institute of Health and Society, Newcastle University, Baddiley Clark Building, Richardson Road, Newcastle upon Tyne, NE2 4AX UK; 2 Fuse, UKCRC Centre for Translational Research in Public Health, Newcastle upon Tyne, UK

**Keywords:** Qualitative, Interview study, Behavioural intervention, Public health, Community based, Management information system, Stakeholder, Data collection, Evaluation, Commissioners, Administration, Data-base

## Abstract

**Background:**

Community based providers are well place to deliver behavioural interventions to improve health. Good project management and reliable outcome data are needed to efficiently deliver and evaluate such interventions, and Management information systems (MIS) can facilitate these processes. We explored stakeholders perspectives on the use of MIS in community based behavioural interventions.

**Methods:**

Stakeholders, purposively selected to provide a range of MIS experience in the delivery of community based behavioural interventions to improve health (public health commissioners, intervention service managers, project officers, health researchers and MIS designers), were invited to participate in individual semi-structured interviews. We used a topic guide and encouraged stakeholders to reflect on their experiences.: Interviews were recorded, transcribed and analysed using five steps of Framework analysis. We applied an agreed coding framework and completed the interviews when no new themes emerged.

**Results:**

We interviewed 15 stakeholders. Key themes identified were: (i) MIS access; (ii) data and its function; (iii) MIS development and updating. Within these themes the different experiences, needs, use, training and expertise of stakeholders and the variation and potential of MIS were evidenced. Interviews advised the need to involve stakeholders in MIS design and development, build-in flexibility to accommodate MIS refinement and build on effective MIS.

**Conclusions:**

Findings advised involving stakeholders, early in the design process. Designs should build on existing MIS of proven utility and ensure flexibility in the design, to incorporate adaptations and ongoing system development in response to early MIS use and evolving stakeholder needs.

**Electronic supplementary material:**

The online version of this article (10.1186/s12889-018-6363-z) contains supplementary material, which is available to authorized users.

## Background

The National Health Service (NHS) Five Year Forward View [1] emphasised the importance of disease prevention and outlined how behavioural interventions can support people to make healthy behaviour choices to improve their health and prevent disease [2]. It is recognised that good project management with reliable outcome data are needed to provide information for policy makers [2] and modern information technology, including access to web-based hosting, can facilitate good administration and robust data collection through well-designed MIS Voluntary and community sector organisations are well place to deliver behavioural health interventions, but [2] good MIS may be difficult to achieve where intervention providers are working on a small scale or to a tight budget.

The design and implementation of health information systems goes beyond the technical aspects [3] of the system and can incur, “unforeseen costs, unfulfilled promises, and disillusionment (Anderson & Aydin 2005, page vii)” [4]. In one conceptual model the technical aspects, design and implementation consequences of a management information system (MIS) are assumed to be within the control of a single organisation, with MIS design responsive to the information needs of that organisation and its management [4]. However, situations regularly occur where MIS are required to function across different organisations and stakeholder groups [5], thus design and implementation should ideally be responsive to multiple management and information needs [6]. For example intervention providers may use MIS for administration, public health commissioners may access MIS data to monitor and commission interventions and researchers may use these data to evaluate intervention effectiveness.

Different evaluation methodologies can be used to examine MIS design and implementation including surveys, observational methods and qualitative interviews [3]. In this study we used qualitative research and individual interviews to investigate the perspectives of stakeholders, who had a range of relevant roles and experience, on the design and use of MIS in delivery and evaluation of behavioural interventions to improve health. We aimed to explore stakeholders perspectives on the use of MIS in delivery and evaluation of community based behavioural interventions and examine their views on how to optimise the design, provision and utility of MIS where these are required to function across different organisations.

## Methods

### Data collection and analysis

To identify stakeholders and recruit study participants we initially focussed on two empirical research studies: ‘Ways to Wellness,’ a social prescribing intervention targeting people in socio-economically deprived communities with long-term health conditions [7, 8] and ‘New life, New you’ a small scale intervention for the prevention of type 2 diabetes in adults at high risk, which included a cultural adaptation of the intervention that was specifically designed to engage local black and ethnic minority communities [9, 10].

#### ‘Ways to Wellness’ social prescribing intervention

Ways to Wellness social prescribing intervention is available to people aged 40 to 74 years, with one or more long-term health condition (diabetes, heart disease, lung disease, osteoporosis, asthma, epilepsy) living in an urban area of high socio-economic deprivation. Patients are referred to the service by their doctor and allocated to a link worker, trained in behavioural techniques, who supports their clients to improve their health behaviours, self-care, condition management and social integration. ‘Ways to Wellness’ takes account of the wider determinants of health and aims to improve clients health related outcomes.

#### ‘New life, New you’ intervention for prevention of type 2 diabetes

‘New life, New you’ behavioural intervention is available to people at high risk of type 2 diabetes, living in an urban area of high socio-economic deprivation. Clients are recruited from the community and there is a specific focus on recruitment of people of Black and UK minority ethnic populations. ‘New life, New you’ is delivered by fitness trainers as group supervised physical activity sessions, with space for reflection, behavioural techniques and nutritional advice. ‘New life, New you’ aims to prevent or delay the onset of type 2 diabetes.

From these studies we identified different stakeholder roles in the provision and evaluation of interventions to improve health as: public health commissioners, voluntary and community sector service managers, project officers (administration and intervention delivery staff), independent researchers and MIS designers. We used purposive sampling to provide a spread of experience across these roles and invited stakeholders to participate in individual semi-structured interviews. Initially we focussed recruitment on the two empirical studies outlined although all study participants had relevant MIS experience beyond these specific studies. To ensure balance across the different stakeholder groups we also accessed other research contacts from within the Institute of Health and Society at Newcastle University. Interviews were conducted face to face or by telephone using a topic guide that covered views on MIS use generally and detailed consideration of MIS use in one or more specific behavioural interventions. All interviews were digitally audio recorded and transcribed for analysis. We used the five steps of Framework analysis: (1. Familiarization, 2. Identifying a thematic framework, 3. Indexing, 4. Charting and 5. Mapping / interpretation), to analyse transcripts and identify anticipated and emergent themes [11, 12]. Researchers LP, LG and AH each coded two different interview transcripts and then met to discuss and agree a coding framework. We used constant comparison to ensure the robustness of the coding framework which was then applied to these six transcripts [13] NVivo 11 software was used to facilitate data management and a further two transcripts were independently coded by LP and AH to further check and validate the framework. We completed the interviews when no new themes emerged from the analysis (data saturation was reached) and the final coding framework was applied to all transcripts.

Researchers LP, LG and AH conducted a total of 15 interviews, (*n* = 6 face to face and *n* = 9 by telephone), between August 2016 and November 2017. Interviews lasted between 20 min and 59 min, with a mean duration of 41 min. Participant roles and demographics are summarised in Table 1.

**Table 1:** Participant demographics

Participant	Role	Age category	Gender
1	Service manager	2	F
2	Project officer	1	F
3	Service manager	2	F
4	Project officer	2	F
5	Commissioner	2	F
6	Commissioner	1	F
7	Project officer	1	M
8	Researcher	1	F
9	Researcher	1	F
10	Commissioner	2	F
11	Database designer	1	F
12	Database designer	2	F
13	Service manager	2	F
14	Commissioner	1	M
15	Service manager	2	F

Age category 1) 20 to 40 years; 2) > 40 years

## Results

From our thematic analysis of interview data we identified three key themes as: (i) access to MIS; (ii) data and its function; (iii) development and updating of MIS. These themes and sub-themes are summarised in Table 2 and then described in detail, with brief supporting quotations below. More detailed quotations are also provided in Additional file 1.

**Table 2:** Summary of themes and sub-themes in thematic analysis

Main themes	Minor categories	Explanation
Access to MIS	Collecting and inputting data	Participants spoke about data collection as a predominantly paper based exercise, although this was seen as pragmatic and cost driven, rather than ideal.
	Setting and time of MIS access	Data entry was usually an office based procedure, whereas review and analysis might rely on remote access. Modern technology facilitated multiple simultaneous use
	Reasons for stakeholder access	Access for data input, administration, monitoring and evaluation was described
	Support and training in MIS use	Both formal and informal training were reported with the need for training to be an ongoing process.
	Usability and value of MIS	Ease of use and ability to accommodate different levels of expertise and confidence were explained.
Data and its function	Confidence in the data	Data quality was a frequent theme that included accurate and secure data with consistency of MIS use, safeguards and ‘back –up’
	Data processing	Data cleaning, extraction, analysis and linkage were explored
	Use of the data	Use of MIS data in administration, monitoring the progress of individual participants and monitoring the progress of the entire project were described.
Development and updating of the MIS	Procurement of the MIS,	The cost complexity and ownership of procured MIS were important aspects of this theme
	Specificity of the MIS, degree to which it is bespoke or generic	Both fairly simple (built on generic platforms) and complex, bespoke MIS were described.
	Stakeholder involvement in the development of the MIS	There was consensus on the need to involve different stakeholders in MIS development from the outset. And the complexity of preserving data integrity within a ‘live’ data collection environment was acknowledged.

### Access to MIS

The key theme of access to MIS encompassed different stakeholder access requirements and included the sub-themes: collecting and inputting data, setting and time of access, reasons for access by different stakeholders, perspectives of MIS value and usability, and support and training in the use of MIS. Each of these sub-themes are detailed, with supporting quotes, below.

#### Collecting and inputting data

Participants spoke about data collection as a predominantly paper based exercise. This was seen as a pragmatic, but not an ideal strategy and there was support for more digitally facilitated data collection.

*“If you worked out how much it cost for an iPad versus having someone's time cost to sit and enter things twice it probably saves money.” (researcher 8)*

Direct input to an electronic system, and a workable strategy for this, was also described.

*“They’ve got laptops, [and] when they’re back at base they can sync it all back up.” (commissioner 14)*

There was concern over the impact of onerous data collection on service user experience, for both paper based and electronic data collection methods.

*“The service user is either going to disengage with the service completely or we are only going to get partial data”. (service manager 15)*

Often those inputting the data were different from those who had collected the data and this could lead to delay and frustration as described below.

*“It [data] will come in in dribs and drabs.” (service manager 3)*

In a different situation the ultimate adverse consequences of a paper based data collection strategy were explained.

*“I think it [data]’s sitting on pieces of paper in a draw somewhere.” (researcher 8)*

In contrast an automated system where intervention participants entered some of their data directly to an online hosting facility was described.

*“You can just give it [tablet] to the patient, they type the stuff in themselves.” (database designer 12)*

#### Setting and time when the MIS was accessed

We asked about when and where stakeholders accessed the MIS and found that this was related to their reason for access. Where MIS access was to enter data, this was usually described as an office based procedure.

However, for data review and analysis, web-hosting facilitated access from other settings, including working from home.

The benefit of new technology, was also described in terms of facilitating multiple simultaneous use. For example an old system might only allow one person to access at any one time whereas a new system could allow multiple access.

*“It’s [MIS] been set up for .. [name] study, where multiple people can be in it [MIS] at once.” (researcher 9)*

#### Access to the MIS, reason for stakeholder access

Stakeholders described their different MIS access needs, knowledge and permissions. Reasons for accessing the MIS were for data input, administration of the intervention programme and for monitoring and evaluation.

Some MIS were set up to alert administrators to non-attendance of intervention clients, which might be linked to applications, such as mail-merge or text messaging to send participant reminders.

*“We want to track as patients drop off the programme.” (service manager 1)*

*“If somebody hasn't attended, we send out a non-attendee letter notifying them.” (commissioner 5)*

In contrast, those responsible for external evaluation might not need access to client contact details and MIS access was set up accordingly as explained in the different levels of access permissions and how these had to be justified.

*“The people pulling it [data] off at the other end, it [data] was completely anonymous.” (service manager 3)*

In one scenario client access to their own data, as a self-regulatory part of the intervention, was described.

*“They [clients] are each provided with a key with a programme on and what it does, it records what they've done. They can compare week by week whether they've improved” (commissioner 5)*

#### Support and training in use of the MIS

Training consisted of both formal and more informal procedures, and the need for training to be an ongoing process was evident. A training pathway, from formal introduction to self-supportive user groups, was described by some participants.

*“We had half a day [training] at the very beginning with everyone who was new … .. then it was about week three or four where we came together for another half a day [training] [Now staff] convene their own user groups … they’re training one another” (service manager 1)*

The collaboration and supportive role of MIS users was a frequent theme.

Some people, especially if they were not involved in all aspect of MIS use, spoke about wanting to understand it better and particularly how an appreciation of different data functions might help stakeholders to appreciate the importance of the data to different end-users.

*It would be good if there was someone like the evaluation team coming in to do something … around, “This is why it's important. This is what a validated measure means.” (project officer 4)*

Others, especially if they had responsibilities for data extraction and analyses spoke about the need for training to support data quality.

*“You can't remove the human factor. I think the staff need continual training. It's hard because some of them have been using this system [MIS] for years, even though it's a new service [intervention] they think they know it all.” (researcher 8)*

#### Usability and value of MIS

Designers of MIS spoke about making the system easy for people to use and the need to accommodate different levels of expertise and confidence in MIS use and the ways in which interface design could make systems better for end-users.

*“You’re going to have people there who are good with computers and people who are like, "I don’t want to touch it. If you can make it [screen] look like what they see in front of them on paper, it’s just better.” (database designer 12)*

However, the value of MIS might not be properly appreciated.

*“The expectation within a project that that it [MIS} is a valued part – an absolutely integral part – of what is being developed, alongside, obviously, the high priority around the actual service that’s being delivered. From my experience, it’s [MIS] never really had a high enough priority.” (service manager 15)*

### Data and its function

This key theme included: confidence in the data (quality, security and accuracy), data processing (cleaning, extraction, analyses and linkage), and use of data (administration, monitoring, evaluation).

#### Confidence in the data, including data quality, security and accuracy

The need for data to be accurate and secure was a frequent theme. Respondents spoke about accuracy in terms of data input, the need to make sure all those inputting data were using the MIS in the same way to ensure consistency*,* and ways in which MIS design could facilitate accuracy in data input. The safeguards to prevent data input error were clearly important for managing quality, for example drop down boxes could improve consistency by limiting field choice. However, mandatory fields (which meant that there was no distinction between a ‘not answered’ and ‘no’ response) were an issue. The quality of self-report data was questioned, with particular reference to a situation where participants reported their own weight over the phone, after a weight loss intervention, and the weight loss was greater than expected.

*“I was thinking, "Well that will explain why the BMI looks so great at follow up.” (project officer 7)*

##### Data security was a major issue, especially where systems included NHS patient data

Details of a security procedure was explained as:

*“ ‘Two-factor authentication’, so two levels of encryption, and two passwords to get in.” ( project officer 2)*

Different levels of access contributed to the data security.

*“We've got reviewer access, which means we can see everything but it's all anonymised.” (researcher 8)*

Also, the need for data ‘back –up’ was seen as important.

*“Having a backup as well … making sure you've got the databases backed up.” (project officer 7)*

#### Data processing, including data cleaning, extraction, analysis and linkage

Ensuring data was ‘cleaned’ and ready for analysis was raised as essentially a planning issue.

*“It can be that the amount of work taken to look over some data, clean it and get it in a nice, presentable fashion, is a lot more than anticipated at the design stages of a study” (database designer 12)*

The difficult of data linkage and*,* “*trying to get different systems to talk to each other” (commissioner 5)* was mentioned. The need to complement pre-set queries with manual calculations was seen as important to identify data trends and one respondent spoke about transferring data to a *‘monitoring and evaluation database’* to follow trends. *(commissioner 5)*

Some of the security issues impacted on data analyses, especially with NHS data. Difficulties with individual level data, even when data were anonymised, were raised.

#### Use of the data, including administration, monitoring and reporting

Data was used for administration purposes, such as sending reminders when people failed to attend the intervention or when they needed to come for their follow-up appointment or review, which often involved collection of outcome data. Monitoring the progress of individual participants was seen as an important use of data, as well as monitoring the progress of the entire project, both in terms of overall participant progress and in comparison with other projects and areas.

*“From the commissioner perspective, it gives them assurance that what they are commissioning is having an impact on the outcomes, the indicators and the performance measures of the services that they’re commissioning.” (commissioner 14)*

### Development and updating of the MIS

Which included: procurement (cost complexity and ownership), specificity (bespoke or generic) and stakeholder involvement (degree of stakeholder input).

#### Procurement of the MIS, including cost complexity and ownership

Procurement rules for public service MIS were mentioned.

The cost of MIS development was a strong theme and there was clearly a balance between ideal and pragmatic solutions.

*“That was a very, very expensive, tailored database. Where the one we’ve got is fine.” (project officer 4)*

Ownership of the MIS was raised by one manager. The intellectual property was not always clear-cut, especially when MIS development had evolved.

*“I think it is our intellectual property, I think anyone who goes into this probably would want to make sure that it is their IP, if they ever need to move it [platform].” (service manage 1))*

#### Specificity of the MIS, degree to which it is bespoke or generic

Stakeholders described MIS that were fairly simple and built on generic platforms, such as Microsoft Access and MIS that were complex and bespoke. There was some support for simple platforms.

*“I think the simple platforms have their place … and probably work well particularly with the volunteering community and social enterprise sector. For them it can provide them with a basis to be able to build up that knowledge of management information system and prove what they’re doing is successful and get them in a position to be able to bid for more work.” (commissioner 14)*

Whereas the problems of a new, complex system were to some extent considered inevitable.

*“There are lots of very legitimate, kind of, bugs you need to work out with a new system” (service manager 1)*

However, participants identified a clear link between size and complexity of service and MIS needed.

The opportunities to co-ordinate services through the use of complex and co-ordinated MIS were described.

*“They [managers] took the ambitious view that what we needed was one [MIS} for [the lead provider organisation] and to be able to layer it so each delivery organisation had the right system, information storage and reports that they needed.” (commissioner 10)*

#### Stakeholder involvement in the development of the MIS

Amongst all respondents, there was consensus about the need to involve different stakeholders in MIS development from the outset.

*“Bring everybody together, the people who are going to be using it every day.” (project officer 2)*

Sometimes stakeholder involvement, although agreed to be desirable, was hampered by practicalities of delivery timescales.

*“I would rather have sat down and done it together, … ... But because our study was very busy, everything was needed now, now, now, so we didn't have that time to be able to do it.” (researcher 9)*

The need for MIS development to be multi-stage was also described along with the tendency for MIS importance to be overlooked.

*“Yes, it definitely is multi-stage. If you develop a database for capture of data for a study, you don’t just build it. You have lots of drafts, and it’s expected that the whole study team could comment and make useful suggestions..” (database designer 12)*

#### How MIS updates are managed

The difficulties of complex MIS were reflected in the many references to ‘updating’ in stakeholder interviews. The need to make the system user friendly was complicated by the need to preserve data integrity within a ‘live’ data collection environment.

*“We have been trying to make it more usable for [delivery staff] but I think it will be ever-evolving, really, to adapt to their needs and to make data entry easier and more efficient for them” (project officer 2)*

The need for in-built flexibility was described, with the facility for stakeholder feedback, but the approach to feedback was seen as variable.

*“You need to have the functionality to continue to grow and develop.” (service manager 13)*

## Discussion

### Main findings

Stakeholders appreciated the value of well-designed MIS and evidenced the need to involve a range of stakeholders, taking into account their different information requirements, to improve MIS design and utility. Training in the use of MIS as an evaluation tool was viewed as important to maximise the accuracy and utility of data collected. The potential for inputting data at the point of patient engagement, thus making use of developments in information technology, was highlighted by stakeholders.

MIS design that builds on existing systems of proven utility, with in-built flexibility to accommodate revisions and refinement in response to early use, was regarded as most likely to provide optimal MIS for delivery and evaluation of community based behavioural interventions.

### Strengths and limitations

The rapid development of information technology means that MIS opportunities are constantly evolving and some of the procedures and perspectives evidenced here, such as a reliance on paper based data collection, may be superseded by new options, including data input by intervention participants. However, the general principles of stakeholder involvement, building on effective systems and providing system flexibility are likely to be resilient to technological innovation.

### Comparison with other studies and implications

In their paper on stakeholder roles and perceptions in health information systems Pouloudi et al. [14] discuss the development and implementation of information systems in the UK National Health Service. They cite a commonly used definition of a stakeholder in relation to an organisation as, ‘*A stakeholder in an organization is (by definition) any group or individual who can affect or is affected by the achievement of the organization’s objectives’* [15] However, Pouloudi et al. then go on to expand this definition, extending the scope and shifting the focus beyond a single organisation, and defining information systems stakeholders as, ‘*The individuals, groups, organizations or institutions who can affect or be affected by an information system.’* [14] This broad definition is applicable to our study, where the intra-organisational use of MIS is the research focus.

At a macro level, the World Health Organization explains the importance of, ‘Sound and reliable information’ as the ‘foundation of decision-making across all health system building blocks’. [16] (WHO report page 1). It is reasonable to assume that information collected locally is similarly important at a local level. There has long been interest in the use of design science to inform information systems [17] and a distinction has been made between natural and social sciences, which have ‘understanding reality’ as their focus, and design science, where the focus is more towards ‘solving problems and making things that are useful in human service’ [18]. In this study we used standard qualitative research techniques and, by understanding different stakeholder perspectives on their experience of MIS, we explored how to optimise the design, provision and utility of these MIS.

### Unanswered questions and future research

The need to shift the health care focus more towards prevention, was identified in the NHS Five Year Forward View [1]. Community based services are well placed to support this shift in focus, but the value of such services cannot be assessed without reliable and accessible information. In particular there is a pressing need to evaluate the effectiveness and cost-effectiveness of preventive interventions to inform decisions on the allocation of resources for health improvement. Any such evaluation relies on the quality and completeness of information and data collection. In turn efficient service administration is needed to facilitate good data collection. Stakeholders interviewed in this study all valued the MIS data, collected through the interventions that they were involved with, for their different information requirements. We suggest further research on use of MIS in community based organisations is needed to inform the efficient development of secure systems with optimal utility. In particular there is a need to explore the linkage between health service (e.g. NHS) data and data collected by community based organisations.

## Conclusion

Well-designed MIS were appreciated by a range of stakeholders, with experience of many projects, for their various information needs. Involving a range of stakeholders, at an early stage in the process, could improve MIS design and training in the use of MIS as an evaluation tool was considered important to maximise data utility.. MIS design that builds on existing systems of proven utility, with in-built flexibility to accommodate revisions and refinement in response to early use, was regarded as most likely to provide optimal MIS for delivery and evaluation of community based behavioural interventions.

## Additional file

Additional file 1: Results with detailed quotations. The appendix provides more detailed results with more extensive quotations. (DOCX 20 kb)

## Abbreviations

MIS: Management Information Systems
NHS: National Health Service in the United Kingdom
