# Supplementary material for: Management information systems for community based interventions to improve health: qualitative study of stakeholder perspectives
Source: BMC Public Health. 2019 Jan 23;19:105. doi: 10.1186/s12889-018-6363-z (PMC6343312; doi:10.1186/s12889-018-6363-z)
Supplement: Supplementary file 1 — Results with detailed quotations. The appendix provides more detailed results with more extensive quotations. (DOCX 20 kb) [file 12889_2018_6363_MOESM1_ESM.docx]

**Appendix 1: Results with detailed quotations**

**Access to MIS**

The key theme of access to MIS encompassed different stakeholder access requirements and included the sub-themes: collecting and inputting data, setting and time of access, reasons for access by different stakeholders, perspectives of MIS value and usability, and support and training in the use of MIS. Each of these sub-themes are detailed, with supporting quotes, below.

***Collecting and inputting data***

Participants spoke about data collection as a predominantly paper based exercise. This was seen as a pragmatic, but not an ideal strategy and there was support for more digitally facilitated data collection.

*“In more cases than not, I think, they [delivery staff] meet with them [clients] and write things in paper copy.” (service manager 1)*

*“In an ideal world, with all the money in the world, they'd all have iPads with the internet on them and that kind of thing. There's just not the resource for that, which is a shame because actually if you worked out how much it cost for an iPad versus having someone's time cost to sit and enter things twice it probably saves money.” (researcher 8)*

Direct input to an electronic system, and a workable strategy for this, was also described.

*“They’ve got laptops, handheld systems, that they can input the data onto and it doesn’t necessarily have to be online, so that basically when they’re back at base they can sync it all back up.” (commissioner 14)*

There was concern over the impact of onerous data collection on service user experience, for both paper based and electronic data collection methods.

*“The service user is either going to disengage with the service completely or we are only going to get partial data around what we want because they are not going to want to sit for a long time providing that data”. (service manager 15)*

Often those inputting the data were different from those who had collected the data and this could lead to delay and frustration as described below.

*“I physically enter that [data] from the paper source onto the database…. When you’re talking about the six month and the twelve month [data collection times] when people are drifting off and away, it [data] won’t all come in in one time. It [data] will come in in dribs and drabs.” (service manager 3)*

In a different situation the ultimate adverse consequences of a paper based data collection strategy were explained.

*“In the end I've had to just give up and write my evaluation report and just say, "I could not get access to this data." I think it's sitting on pieces of paper in a draw somewhere. (researcher 8)*

In contrast an automated system where intervention participants entered some of their data directly to an online hosting facility was described.

*“Other information systems definitely do use tablets because you can just give it to the patient, they type the stuff in themselves and then there isn’t anyone required to type it up. To be honest, that would be great for us.” (database designer 12)*

***Setting and time when the MIS was accessed***

We asked about when and where stakeholders accessed the MIS and found that this was related to their reason for access. Where MIS access was to enter data, this was usually described as an office based procedure.

*“I think they’re all at laptops or desktops in their team offices, when they do their [data] entry.” (service manager 1)*

However, for data review and analysis, web-hosting facilitated access from other settings, including working from home

*“I do have quite a few occasions where I’ll work from home…. I would say that, every day, I would be logging onto the MIS and looking at various, different sets of data.” (project officer 2)*

The benefit of new technology, was also described in terms of facilitating multiple simultaneous use. For example an old system might only allow one person to access at any one time whereas a new system could allow multiple access.

*We had it [database] just in a shared drive. Now, I know it's [new database] been set up for .. [name] study, where multiple people can be in it [database] at once.” (researcher 9)*

***Access to the MIS, reason for stakeholder access***

Stakeholders described their different MIS access needs, knowledge and permissions. Reasons for accessing the MIS were for data input, administration of the intervention programme and for monitoring and evaluation.

*“It’s mainly me who uses it, yes, so I’m the one entering all the details in, all the update details, and pulling the information off as required.” (project officer 4)*

Some MIS were set up to alert administrators to non-attendance of intervention clients, which might be linked to applications, such as mail-merge or text messaging to send participant reminders.

*“We want to track as patients drop off the programme.” (service manager 1)*

*“If somebody hasn't attended, we send out a non-attendee letter notifying them.” (commissioner 5)*

In contrast, those responsible for external evaluation might not need access to client contact details and MIS access was set up accordingly as explained in the different levels of access permissions and how these had to be justified.

*“The people pulling it [data] off at the other end, it [data] was completely anonymous. (service manager 3)*

*“We have to write a very clear justification of why we’d need to see that level [participant personal details] of information”. (commissioner 14)*

In one scenario client access to their own data, as a self-regulatory part of the intervention, was described.

*“They [clients] are each provided with a key with a programme on and what it does, it records what they've done. They can compare week by week whether they've improved, different things like heart rate, calories and what steps they've done, how many minutes they've exercised for. It's quite a useful tool for them because they tend to monitor themselves as well and see how they're getting on” (commissioner 5)*

***Support and training in use of the MIS***

Training consisted of both formal and more informal procedures, and the need for training to be an ongoing process was evident. A training pathway, from formal introduction to self-supportive user groups, was described by some participants.

*“We had half a day [training]at the very beginning with everyone who was new….. Then, they [delivery staff] got a chance to use it [MIS], and referrals were starting to come in. Like, the service was live, and then it was about week three or four where we came together for another half a day [training]. But, we haven’t had anything since except to have, kind of, them [staff] convene their own user groups … they’re training one another” (service manager 1)*

The collaboration and supportive role of MIS users was a frequent theme.

*“If it was something that I wasn't comfortable with, I would just ask the appropriate person for some support on it” (commissioner 5)*

Some people, especially if they were not involved in all aspect of MIS use, spoke about wanting to understand it better and particularly how an appreciation of different data functions might help stakeholders to appreciate the importance of the data to different end-users.

*“I’d like to understand it more. I’d have to have somebody talk me through it and I am sure I would be fine but it is just the time.” (service manager 3)*

*“The training that I went on, which is what the staff get, is "This is literally how you input data into an online system." It would be good if there was someone like the evaluation team coming in to do something alongside that around, "This is why it's important. This is what a validated measure means." (project officer 4)*

Others, especially if they had responsibilities for data extraction and analyses spoke about the need for training to support data quality.

*“You can't remove the human factor. I think the staff need continual training. It's hard because some of them have been using this system [MIS] for years, even though it's a new service [intervention] they think they know it all.” (researcher 8)*

***Usability and value of MIS***

Designers of MIS spoke about making the system easy for people to use and the need to accommodate different levels of expertise and confidence in MIS use and the ways in which interface design could make systems better for end-users.

*“You’re going to have people there who are good with computers and people who are like, “I don’t want to touch it.” Those are the people that ring you and say, “The database is doing something funny.” You’re like, “No, you’re just doing something-” and you have to talk them through. If you can make it [screen] look like what they see in front of them on paper, it’s just better.” (database designer 12)*

MIS users explained the value and the opportunities such systems could provide.

*“There wouldn't really be another way for us to get data from 3000 people and have it all stored neatly in one place” (researcher 8)*

However, the value of MIS might not be properly appreciated.

*“The expectation within a project that that it [MIS} is a valued part – an absolutely integral part – of what is being developed, alongside, obviously, the high priority around the actual service that’s being delivered. From my experience, it’s [MIS] never really had a high enough priority.” (service manager 15)*

**Data and its function**

This key theme included: confidence in the data (quality, security and accuracy), data processing (cleaning, extraction, analyses and linkage), and use of data (administration, monitoring, evaluation)

***Confidence in the data, including data quality, security and accuracy***

The need for data to be accurate and secure was a frequent theme. Respondents spoke about accuracy in terms of data input, the need to make sure all those inputting data were using the MIS in the same way to ensure consistency*,* and ways in which MIS design could facilitate accuracy in data input. The safeguards to prevent data input error were clearly important for managing quality, for example drop down boxes could improve consistency by limiting field choice. However, mandatory fields (which meant that there was no distinction between a ‘not answered’ and ‘no’ response) were an issue. The quality of self-report data was questioned, with particular reference to a situation where participants reported their own weight over the phone, after a weight loss intervention, and the weight loss was greater than expected.

*“I was thinking, "Well that will explain why the BMI looks so great at follow up.” (project officer 7)*

Data security was a major issue, especially where systems included NHS patient data, and specific security procedures described.

*“They [NHS regulatory body] were very concerned that we made sure we followed the information and governance that was required.” (project officer 2)*

Details of a security procedure was explained as:

*“ ‘Two-factor authentication’, so two levels of encryption, and two passwords to get in.” ( project officer 2)*

Different levels of access contributed to the data security.

*“It's an online NHS system, so it's all very secure, and me and the rest of the evaluation team have access but you can get different levels of access. We've got reviewer access, which means we can see everything but it's all anonymised.” (researcher 8)*

Also, the need for data ‘back –up’ was seen as important.

*“Having a backup as well really. It's just developing that as well, making sure you've got the databases backed up.” (project officer 7)*

***Data processing, including data cleaning, extraction, analysis and linkage***

Ensuring data was ‘cleaned’ and ready for analysis was raised as essentially a planning issue.

*“It can be that the amount of work taken to look over some data, clean it and get it in a nice, presentable fashion, is a lot more than anticipated way at the design stages of a study” (database designer 12)*

The difficult of data linkage and *, “ trying to get different systems to talk to each other” (commissioner 5)* was mentioned. The need to complement pre-set queries with manual calculations was seen as important to identify data trends and one respondent spoke about transferring data to a *‘monitoring and evaluation database’* to follow trends. *(commissioner 5)*

Some of the security issues impacted on data analyses, especially with NHS data. Difficulties with individual level data, even when data were anonymised, were raised.

***Use of the data, including administration, monitoring and reporting***

Data was used for administration purposes, such as sending reminders when people failed to attend the intervention or when they needed to come for their follow-up appointment or review, which often involved collection of outcome data. Monitoring the progress of individual participants was seen as an important use of data, as well as monitoring the progress of the entire project, both in terms of overall participant progress and in comparison with other projects and areas.

*“So, we can see what progress we’re making and whether they’re dropping out, whether they’re continuing with the programme, and assess how that is working. (service manager 3)*

*“We can do then benchmarking with our neighbouring authorities or other authorities that have got a similar demographic makeup to ours.” (commissioner 6)*

*“From the commissioner perspective, it gives them assurance that what they are commissioning is having an impact on the outcomes, the indicators and the performance measures of the services that they’re commissioning.” (commissioner 14)*

**Development and updating of the MIS**

Which included: procurement (cost complexity and ownership), specificity (bespoke or generic) and stakeholder involvement (degree of stakeholder input)

***Procurement of the MIS, including cost complexity and ownership***

Procurement rules for public service MIS were mentioned.

*“Any NHS foundation trust, so any hospital setting would have to have an information system. To enable them to get that information system, they would have to go out to tender because of the value of the contract.” (commissioner 6)*

The cost of MIS development was a strong theme and there was clearly a balance between ideal and pragmatic solutions.

*“That was a very, very expensive, tailored database. Where the one we’ve got is fine, it’s not as sophisticated. Obviously, you’re talking a lot more money to be able to maintain something like that.” (project officer 4)*

*“I did a little study .. and for that I setup my own database. I knew exactly what I wanted, and I knew exactly what I wanted it to do, and I was in complete control of it.” (database designer 11)*

Ownership of the MIS was raised by one manager. The intellectual property was not always clear-cut, especially when MIS development had evolved.

*I think it is our intellectual property, but I think that’s important for… We’re going to have to find that out explicitly, and I think anyone who goes into this probably would want to make sure that it is their IP, if they ever need to move it [platform].” (service manage 1))*

***Specificity of the MIS, degree to which it is bespoke or generic***

Stakeholders described MIS that were fairly simple and built on generic platforms, such as Microsoft Access and MIS that were complex and bespoke. There was some support for simple platforms.

*“I think the simple platforms have their place and they can work well, and probably work well particularly with the volunteering community and social enterprise sector. For them it can provide them with a basis to be able to build up that knowledge of management information system and prove what they’re doing is successful and get them in a position to be able to bid for more work.” (commissioner 14)*

Whereas the problems of a new, complex system were to some extent considered inevitable.

*“There are lots of very legitimate, kind of, bugs you need to work out with a new system” (service manager 1)*

However, participants identified a clear link between size and complexity of service and MIS needed.

*“The bigger the numbers you are getting through the more you need a system that organises it all” (service manager 3)*

*“We thought that if we could build something bespoke, and it could be all digital. We would have a lot of power, having all that data, to be able to analyse it, have it be real-time.” (service manager 1)*

The opportunities to co-ordinate services through the use of complex and co-ordinated MIS were described.

*“They [managers] took the ambitious view that what we needed was one [MIS} for [the lead provider organisation] and to be able to layer it so each delivery organisation had the right system, information storage and reports that they needed. (commissioner 10)*

***Stakeholder involvement in the development of the MIS***

Amongst all respondents, there was consensus about the need to involve different stakeholders in MIS development from the outset.

“Bring everybody together, the people who are going to be using it every day. Have a group that involves your stakeholders, so you fully understand what they expect from the MIS or what reports they’re going to want from you, and then you can fulfil those requirements.” *(project officer 2)*

*“What we did at the start was sit down with the service and make clear what we needed for our evaluation. “ (researcher 8)*

Sometimes stakeholder involvement, although agreed to be desirable, was hampered by practicalities of delivery timescales.

*“I would rather have sat down and done it together, just because I think it's really important for the people that are using it to know what those initial stages are and how it was set up, and I think that would've definitely helped. But because our study was very busy, everything was needed now, now, now, so we didn't have that time to be able to do it.” (researcher 9)*

The need for MIS development to be multi-stage was also described along with the tendency for MIS importance to be overlooked.

*“Yes, it definitely is multi-stage. If you develop a database for capture of data for a study, you don’t just build it. You have lots of drafts, and it’s expected that the whole study team could comment and make useful suggestions. Sometimes, there’s a reluctance to engage because it’s seen as, “That’s just the data stuff. Someone else deals with that. That’s not important. Really, when it comes to the end of the study and a variable is missing that they wanted to analyse, you, sort of- yes, there needs to be a bit more foresight, I think, around data management as a whole.” (database designer 12)*

***How MIS updates are managed***

The difficulties of complex MIS were reflected in the many references to ‘updating’ in stakeholder interviews. The need to make the system user friendly was complicated by the need to preserve data integrity within a ‘live’ data collection environment.

*“We have been trying to make it more usable for [delivery staff] but I think it will be ever-evolving, really, to adapt to their needs and to make data entry easier and more efficient for them” (project officer 2)*

*“ Then, that amount of data, if you were to change some field and define it in a different way, does all the old data become incorrect, or at least it’s apples and oranges, you can’t compare old data with new data in that particular domain?” (service manager 1)*

*“We made tweaks along the way….. But the tweaks that we did make were more on the practical side of the data, how we collected it and whether it was needed.” (service manager 3)*

The need for in-built flexibility was described, with the facility for stakeholder feedback, but the approach to feedback was seen as variable.

*“You need to have the functionality to continue to grow and develop.” (service manager 13)*

*With any other MIS system that I’ve come into contact with over the last 20 years, there has been the facility to be able to feed back. Whether that feedback is actually used: that’s been a range of experiences from no response at all to significant work carried out in response to stakeholder feedback. (service manager 15)*
